# CELL REJUVENATION AND DISEASE

**DOI:** 10.1093/geroni/igae098.0868

**Published:** 2024-12-31

**Authors:** Juan Carlos Izpisua Belmonte

**Affiliations:** Altos Labs, San Francisco, California, United States

## Abstract

Aging is characterized by the functional decline of tissues and organs and the increased risk of aging-associated disorders. Different rejuvenating interventions have been proposed to delay aging and the onset of age-associated decline and disease to extend healthspan and lifespan. These interventions include metabolic manipulation, heterochronic parabiosis, pharmaceutical administration and senescent cell ablation. As disease and aging are associated with altered epigenetic mechanisms of gene regulation, such as DNA methylation, histone modification and chromatin remodelling, and non-coding RNAs, the manipulation of these mechanisms might be central to the effectiveness of treating disease as well as age-delaying interventions. I will discuss some of the epigenetic changes that occur during disease and aging and how partial reprogramming by the Yamanaka factors might be a potential avenue to restore cell health and resilience through cellular rejuvenation programming to reverse disease, injury, and the disabilities that can occur throughout life.

